# Buruli Ulcer in Ghana: Results of a National Case Search

**DOI:** 10.3201/eid0802.010119

**Published:** 2002-02

**Authors:** George Amofah, Frank Bonsu, Christopher Tetteh, Jane Okrah, Kwame Asamoa, Kingsley Asiedu, Jonathan Addy

**Affiliations:** * Ministry of Health, Accra, Ghana; † Buruli Ulcer Global Initiative, World Health Organization, Geneva, Switzerland; ‡ University of Ghana Medical School, Accra, Ghana

**Keywords:** Buruli ulcer, epidemiology, case search

## Abstract

A national search for cases of Buruli ulcer in Ghana identified 5,619 patients, with 6,332 clinical lesions at various stages. The overall crude national prevalence rate of active lesions was 20.7 per 100,000, but the rate was 150.8 per 100,000 in the most disease-endemic district. The case search demonstrated widespread disease and gross underreporting compared with the routine reporting system. The epidemiologic information gathered will contribute to the design of control programs for Buruli ulcer.

Buruli ulcer disease is assuming public health importance in many countries, prompting the establishment of a Global Buruli Ulcer Initiative by the World Health Organization (WHO) in early 1998. Ever since *Mycobacterium ulcerans* infection was first described in Australia in 1948 (*1*) and later named Buruli ulcer in Uganda (*2*), cases have been reported throughout the tropical and subtropical world. In the African WHO region, at least 16 of 46 member countries report cases, especially in West Africa and parts of Eastern and Central Africa (*3*).

One characteristic of the disease is its apparent association with bodies of water worldwide (*4*,*5*). The recent identification of *M. ulcerans* in certain water insects has raised the possibility of mechanical transmission of the infection (*6*).

Buruli ulcer commonly affects the young, even though cases are reported in all age groups (*7*,*8*). Oluwasanmi et al. (*5*) and van der Werf (*8*) did not find any sex difference in their series, but Barker reported prevalence to be higher among women than men and among boys than girls. The disease is characteristically found more often on the extremities than on the trunk (*9*). The infection is usually restricted to relatively small areas and patchy in its distribution (*10*,*11*).

The first probable case of Buruli ulcer in Ghana was reported in the Greater Accra Region in 1971; the presence of additional cases along the tributaries of the Densu River in the area was considered likely (*12*). In 1989, van der Werf et al. described 96 cases in the Asante Akim North District of Ashanti Region (*8*). This report was followed by the description of a major endemic focus in Amansie West District in the same region (*13*). Since then, isolated cases have been found in scattered communities in many parts of the country, generating much political and media concern and interest.

In 1993, a passive surveillance system for reporting Buruli ulcer was initiated in Ghana. By the end of 1998, approximately 1,200 cases had been reported from four regions. Gross underreporting was suspected, however, as the media continued to report cases in remote rural communities. Because most cases were known to be in relatively deprived, inaccessible areas, the routine reporting system was judged inadequate to provide a true picture of the extent of disease and the geographic distribution of cases for design of a national control program. In addition, a case search would provide baseline data against which intervention measures could be assessed.

The main objective of the national case search was to establish the extent of the disease in Ghana to facilitate development of a national program for its control. The specific objectives were to determine the epidemiologic characteristics of Buruli ulcer in Ghana and determine physical accessibility of disease-endemic communities to health-care services.

## Methods

### Definition of variables

Geographic distribution was defined in terms of regional, district, subdistrict, and community distribution of cases. The burden of disease was considered in terms of number of cases affected, age and sex distribution, clinical presentation (preulcerative, ulcer, or deformity), and site of lesion. Preulcerative lesions include nodular, plaque, papular, and nonulcerative edematous forms, as described by the WHO Global Buruli Ulcer Programme (*3*). Deformities include scars, constriction of limbs, ankylosis of joints, or amputations.

The case search covered every district and known community in Ghana from June to July 1999. A team of 20 national facilitators was trained in the use of the survey instruments and in the clinical presentation of the disease in an endemic focus. Two facilitators were then sent to each region to train regional teams (three from the regional level and two from each district). Seven teams of two persons each from the subdistrict and communities performed the case search.

The permission of the local political and traditional authorities was sought in advance, and the purpose of the search was explained to them and to all participants. The data collectors used a pictorial document designed by the WHO Global Buruli Ulcer Initiative (*3*). At each village and community, they showed the pictures of Buruli ulcer disease at different stages of development to as many people as possible and asked whether anyone in the village had a similar condition. All persons with lesions that met the WHO standard case definition were interviewed with a simple questionnaire. There was no laboratory confirmation of the cases. A prepackaged dressing was given to each person identified as having ulcers, and the particulars of all the cases were provided to local health authorities for follow-up. The process was repeated in each village until the whole district was covered.

A team from the national level, including a dermatologist familiar with the disease, later validated findings in two randomly selected districts from a region where the disease had not previously been endemic. All cases reported there were found to be consistent with the clinical case definitions used.

### Data Entry and Analysis

Data from all the regions were entered centrally and later cross-checked and edited by EpiInfo 6 software. Data analysis was done both manually and by EpiInfo 6, as appropriate. Yates corrected chi-square and Cornfield 95% confidence interval tests were used as the statistical tests.

Information on regional population distribution was taken from the 2000 national census. About 200 forms from Atwima District of Ashanti Region had to be excluded from the data analysis because information was incomplete on almost all variables. Records with missing data for a particular variable were also excluded from analysis of that variable.

## Results

**Figure 1:**
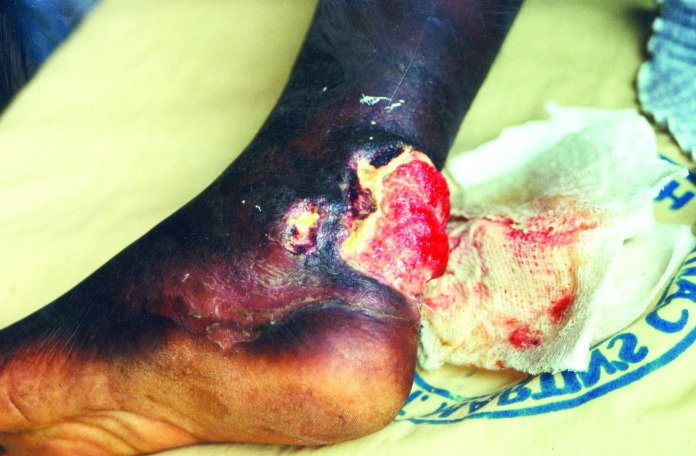
Buruli ulcer on left ankle.

**Figure 2:**
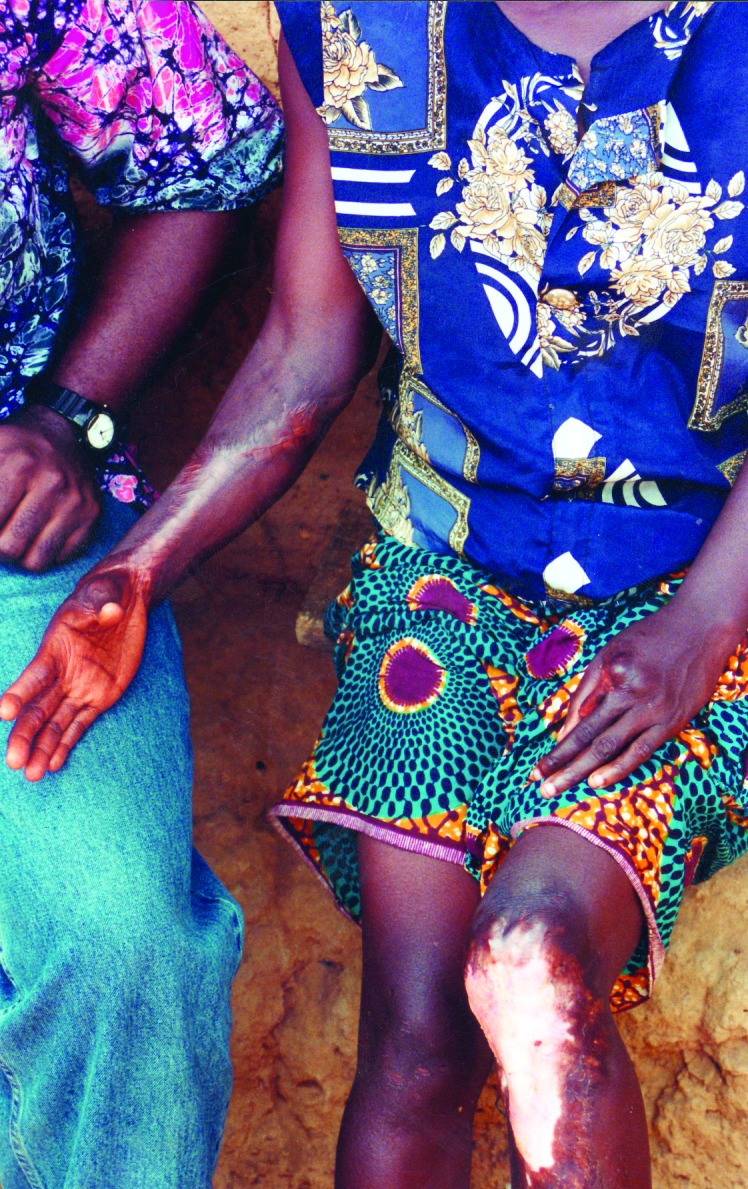
Healed Buruli lesions with scarring, right forearm and left knee.

We identified 5,619 patients with 6,332 suspected Buruli lesions at various stages of development. Approximately 48.5% of the lesions were at the ulcerative stage (Figure 1) and 12.5% at the preulcerative stage; 36.3% had formed scars (Figure 2), and 2.7% of lesions were associated with other forms of deformities.

Of the 5,619 patients, 46% had ulcers only, 10.8% had preulcerative lesions only, and 33.4% had healed scars only. Lesions at both ulcerative and preulcerative stages were seen in 2.5% of the patients; the rest had various combinations of lesions. If only active lesions (preulcerative or ulcerative) were used to calculate the prevalence of the disease in Ghana, 3,725 of the patients (66.3%) had active lesions and 1,894 (33.7%) had healed lesions, for a national crude prevalence rate of 20.7 per l00,000.

### Age and Sex Distribution

Approximately 49% of all patients for whom data were available (n=5,596) were female. The ages of those with active lesions ranged from 0.8 to 100 years (median 25 years; first quartile 12 years; third quartile 50 years; and mean 32 years.)

Among patients with active lesions, age is significantly associated with sex. There are significantly more females than males among patients ≥20 years than those <20 years old (chi square = 14.9; p=0.0001; odds ratio [OR]=1.3; 95% confidence interval [CI] 1.13-1.48). The age-specific odds ratio for male likelihood of having an active Buruli lesion is 0.58 (CI 0.5-0.68; p=0) while that for females is 0.73 (CI 0.62-0.68; p=0.0002).

Of 5,772 lesions for which the information was available, 25.1% were located on the arms and hands, 65.6% on the legs and feet, 5.4% on the trunk, and 3.8% on the head and neck. The distribution of lesions on the limbs (upper or lower limbs) is significantly associated with the age of the patient. Patients >20 years of age have more lesions on the limbs than the younger ones (chi square=16.4; p=0.0001; OR= 1.43; 95% CI 1.20-1.72).

The distribution of lesions on the trunk and lower limbs did not differ by sex. Males, however, had significantly more lesions on the head and neck than females (chi square=6.71; p=0.006; OR=0.67; 95% CI 0.51-0.89), while females had significantly more lesions on the upper limbs than males (chi square=4.56; p=0.03; OR=1.16; 95% CI 1.01-1.33).

Among females, 64.3% of lesions were on the lower limbs, 27% were on the upper limbs, 5.5% on the truck, and 3.2% on head and neck. Among males, 67% of lesions were on the limbs, 23% were on the upper limbs, 5.6% on the trunk and 4.2% on head and neck.

**Figure 3:**
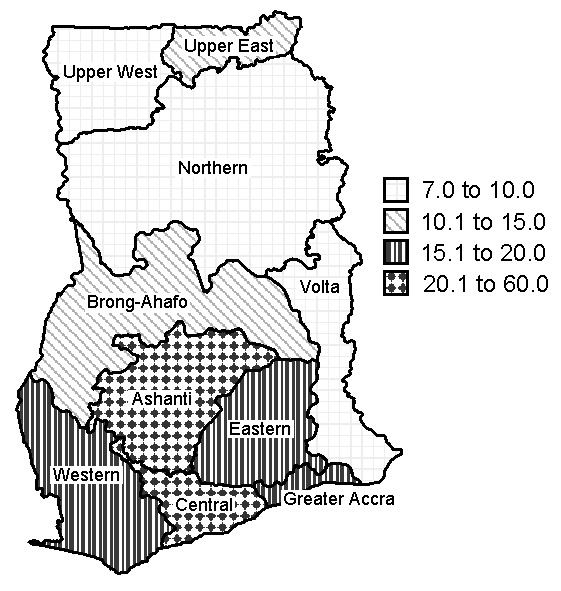
Prevalence of suspected active cases of Buruli ulcer, by region, Ghana, 1999.

Cases of Buruli ulcer were identified in all 10 regions. Table 1 shows the prevalence rates per region, based on estimated 1999 population figures from the 2000 census. The Central Region has the highest overall prevalence rate of active cases, followed by the Ashanti Region; the Northern and Upper West Regions had the lowest prevalence rates (Figure 3). Cases of the disease were identified in 90 (81.8%) of Ghana’s 110 districts. Table 2 shows the prevalence rates of the disease in the 10 districts with the highest caseloads. Amansie West had the highest rate (prevalence 150.8 per 100,000), followed by Asante Akim North (prevalence 131.5 per 100,000) and Upper Denkyira (prevalence 114.7 per 100,000).

**Table 1:** Prevalence of active Buruli ulcer cases by region in Ghana, 1999

Region	No. of males	No. of females	Total no. of active cases	Prevalence (rate per 100,000)
Ashanti	482	475	957	30.8
Brong Ahafo	113	110	223	12.5
Central	519	395	914	59.2
Eastern	202	150	352	16.9
Greater Accra	259	255	514	18.5
Northern	65	68	133	7.4
Upper East	34	63	97	10.7
Upper West	21	21	42	7.4
Volta	78	74	152	9.6
Western	181	140	321	17.9
Unknown			20	
Ghana	1,751	1,954	3,725	20.7

**Table 2:** Prevalence of Buruli ulcer in the 10 districts with the highest caseloads, Ghana, 1999

District	Region	No. of active cases	No. of active and healed lesions	Prevalence (rate of active cases per 100,000)
Ga	Greater Accra	467	1,113	87.7
Amansie West	Ashanti	159	474	150.8
Assin	Central	159	173	83.7
Gomoa	Central	158	161	81.9
Asante Akim N	Ashanti	138	265	131.5
Wassa Amenfi	Western	136	167	61.1
Kwawu South	Eastern	122	132	57.0
Upper Denkyira	Central	121	306	114.7
Afigya Sekyere	Ashanti	118	149	107.1
North Tongu	Volta	107	129	85.7

With regard to access to health care, 38.0% of patients lived in communities >5 miles from the nearest health facility, 37.1% lived 1 to 5 miles away, and 24.9% lived within a mile of the nearest health facility.

## Discussion

All cases were diagnosed on the basis of clinical case definitions without laboratory confirmation; as a result, atypical cases such as early and healed lesions may be confused with other diseases endemic in Ghana (e.g., yaws). Experience, however, shows that in disease-endemic communities Buruli ulcer is readily diagnosed empirically.

The overall crude prevalence rate of 20.7 per 100,000 exceeds that of for leprosy (9 per 100,000) in 1999, making Buruli ulcer the second most prevalent mycobacterial disease in Ghana after tuberculosis (prevalence 66 per 100,000). Before the case search, a cumulative total of approximately 1,200 cases of Buruli ulcer had been reported from five regions in Ghana over a 6-year period from 1993 to 1998. The case search has confirmed that the disease is grossly underreported. The distribution of the disease is much more widespread than earlier thought; suspected cases were identified in all 10 regions and at least 90 of 110 districts. Although the infection was thought to be restricted to relatively small patchy isolated foci separated by large disease-free areas (*11*), our impression is that the more one looks for the disease in known disease-endemic and nearby areas the more likely additional cases will be found.

Our study confirms findings elsewhere that the disease affects children more than adults (*7*,*8*). Marston et al. found the highest rate of infection among children 10-14 years of age in a disease-endemic area in the Daloa Region of Côte d’Ivoire (*14*). This observation has been misinterpreted to mean that the disease affects only children. Our study demonstrates that all age groups can be affected. At least 25% of persons with active lesions were >50 years old.

The preponderance of lesions on the extremities is once again confirmed by our study. About 92% of lesions were located on the extremities in the study by Marston et al. in Côte d’Ivoire (*14*), compared with 91% in our study. Barker noted that among girls and women there was equal frequency of arm and leg lesions, while among boys leg lesions predominate (*9*). Our results, however, show that lesions on the leg predominate for all age groups and both sexes. Even for females, leg lesions were 2.4 times more frequent than arm lesions.

At least 40% of patients with the disease lived in communities ≥5 miles from the nearest health facility capable of providing the minimum service of wound dressing. This distance poses problems for patients who have to travel repeatedly for care for such a chronic ailment.

The study has shown that Buruli ulcer disease in Ghana is much more widespread than previously thought. In all areas where Buruli ulcer cases have been identified, the extent of the disease is likely to be much greater than currently recognized through the routine reporting system. The data set on the disease from the study is among the largest anywhere in the world and can contribute substantially to the epidemiologic description of this relatively new disease. The information generated should contribute greatly to the design, implementation, and evaluation of Buruli ulcer control programs in Ghana.
